# Mechanisms and Target Parameters in Relation to Polycystic Ovary Syndrome and Physical Exercise: Focus on the Master Triad of Hormonal Changes, Oxidative Stress, and Inflammation

**DOI:** 10.3390/biomedicines12030560

**Published:** 2024-03-01

**Authors:** Csanád Endre Lőrincz, Denise Börzsei, Alexandra Hoffmann, Csaba Varga, Renáta Szabó

**Affiliations:** Department of Physiology, Anatomy, and Neuroscience, Faculty of Science and Informatics, University of Szeged, H-6726 Szeged, Hungary; lendre1992@yahoo.com (C.E.L.); borzseidenise@gmail.com (D.B.); hoffmannalexandra1228@gmail.com (A.H.); vacs@bio.u-szeged.hu (C.V.)

**Keywords:** polycystic ovary syndrome, oxidative stress, inflammation, hormonal imbalance, exercise

## Abstract

Polycystic ovary syndrome (PCOS) is a common endocrine disorder among females of reproductive age with heterogeneous prevalence. It is well known that female reproductive competence depends on the dynamic regulation of the hypothalamic–pituitary–gonadal (HPG) axis; therefore, disruption of this highly regulated system leads to fertility problems. Among disruptors, both oxidative stress and inflammation contribute to an increased LH-FSH ratio and a consequent hyperandrogenism. Shifts in this bidirectional interplay between the neuroendocrine system and oxidative/inflammatory homeostasis result in the accumulation of reactive oxygen/nitrogen species and inflammatory markers as well as alterations in antioxidant defense mechanisms. Evidence shows that lifestyle changes, including regular physical exercise, are recognized as the most effective first-line management to reduce the severity of PCOS symptoms. The aim of our narrative review is to provide insights into the mechanisms and target factors of PCOS-related hormonal changes, oxidative/antioxidant homeostasis, and inflammation, and to discuss the effects of exercise, which takes into account various factors, in relation to PCOS. A better understanding of the PCOS-associated hormonal changes, oxidative and inflammatory circuits, as well as exercise-induced mechanisms of action on those targets may improve the quality of life of women with PCOS.

## 1. Introduction

### Background

Polycystic ovary syndrome (PCOS) is one of the most common endocrine disorders among females of reproductive age, whose multifaceted nature has baffled many researchers and clinicians until now. According to the World Health Organization, PCOS affects an estimated 8–13% of reproductive-aged women; however, up to 70% of affected women remain undiagnosed [1].

PCOS is characterized by polycystic ovarian morphology, dysregulated ovarian cycles with a consequent anovulation as well as biochemical and/or clinical hyperandrogenism [2,3]. Neuroendocrine alterations in PCOS can be associated with an increased gonadotropin-releasing hormone (GnRH) pulsatility and an increased luteinizing hormone (LH) secretion from the pituitary gland, which result in an elevated androgen synthesis in the ovarian theca cells [4].

Polycystic ovarian morphology can be associated with changes in follicle pattern. Androgen excess enhances the recruitment of primordial follicles into the pool of growing small pre-antral and antral follicles and impairs selection for dominant follicles. These alterations account for ovarian enlargement, capsular thickening, as well as thecal/stromal hyperplasia and luteinization [5]. In addition to hormonal and histopathologic changes, the hallmark features of this complex endocrinopathy include cutaneous manifestations (e.g., acne, seborrhea, hirsutism, male-pattern alopecia, and virilization), infertility, and large-scale metabolic pathologies, including obesity, hyperinsulinemia, insulin resistance, metabolic syndrome, and type 2 diabetes (T2DM) [6].

**Figure 1:**
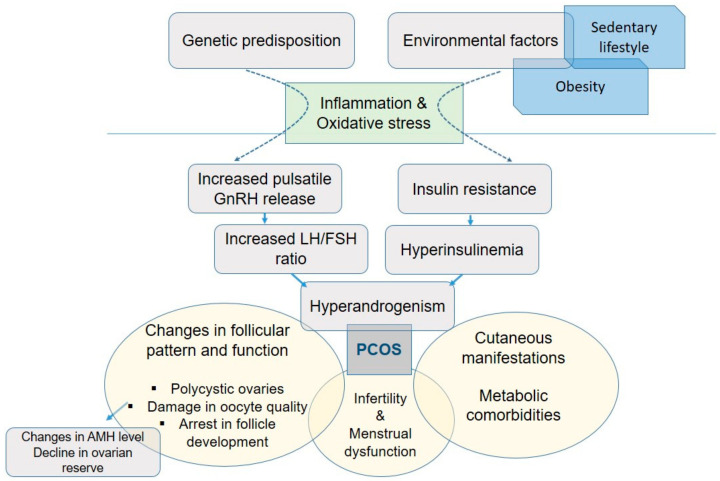
Summarized scheme regarding the pathophysiology of polycystic ovary syndrome. AMH: anti-Müllerian hormone; FSH: follicle-stimulating hormone; LH: luteinizing hormone; PCOS: polycystic ovary syndrome.

Although morphological and endocrine signaling mechanisms are well described in this complex endocrine pathology, the underlying biochemical and molecular mechanisms are not fully elucidated. A growing number of studies examine the role of oxidative/nitrosative stress and inflammatory processes in the development and progression of PCOS [7,8]. During oxidative stress, oxidative/antioxidant homeostasis is disrupted, which results in an accumulation of reactive oxygen species (ROS) and shifts in antioxidant defense mechanisms [9,10,11]. In addition to the accumulation of oxidative stress parameters, increased levels of inflammatory markers and the consequent low-grade inflammation along with hyperandrogenism maintain an unfavorable homeostatic profile in PCOS phenotypes [12]. On that basis, PCOS seems to be a complex trait in which morphological and hormonal features are closely related to inflammation and oxidative stress. Previous studies proved that altered levels of pro-inflammatory cytokines and oxidative stress in the follicular environment can negatively influence ovarian morphology and function [13,14]. Follicular fluid is derived from blood and tissue fluid and contains infiltrated macrophages and lymphocytes. The release of inflammatory and oxidative markers has a negative impact on folliculogenesis and can result in ovarian fibrosis. Obesity and insulin resistance have been implicated as aggravating factors in the pathogenesis of PCOS. Hyperinsulinemia increases androgen production, which promotes adipocyte differentiation and higher free fatty acid levels, thus leading to inflammation, oxidative stress, and a consequent damage in oocyte quality [13]. Figure 1 shows insight into the pathogenesis of PCOS.

Although PCOS is difficult to cure completely, it can be effectively managed. According to the Recommendations of the International Evidence-Based Guideline 2023 for the Evaluation and Management of Polycystic Ovary Syndrome, women with PCOS should make lifestyle interventions, such as performing regular exercise or eating a healthy diet combined with exercise in order to improve quality of life [15]. A systematic review in relation to physical activity and PCOS concluded that the optimal exercise volume, intensity, and duration cannot be fully identified, but a minimum duration of 30 min at a submaximal heart rate level has been shown to improve reproductive functions (the average duration was 15 weeks) [16]. Similar to other non-communicable diseases, physical exercise exerts a protective role in reproductive function and its comorbidities; however, the effects mediated by exercise are largely determined by various factors (e.g., duration and intensity of exercise or the individual overall health status).

Due to the high heterogeneity of PCOS, understanding its underling mechanisms and identifying potential therapeutic interventions are at the heart of research. The purpose of this narrative review is to highlight the role of oxidative stress and inflammation, accompanied by hormonal changes, in the pathogenesis of PCOS. In addition, our aim was to describe the relationship between these PCOS-related changes and physical exercise, focusing on the mechanisms of action and main targets. Understanding these underlying mechanisms may improve the quality of life of women with PCOS and can help to reduce the PCOS-associated comorbidities.

## 2. Hormonal Changes in Patients with PCOS

### 2.1. Hormones of the HPG Axis and PCOS

During the menstrual cycle, androgen and ovarian hormone production is maintained and regulated by upstream signals from the hypothalamic region of the brain through the so-called hypothalamic–pituitary–gonadal (HPG) axis [17]. Neurons in the hypothalamus secrete pulsatile GnRH into the portal vasculature of the pituitary gland. GnRH is responsible for the production and release of gonadotropins, out of which two important ones worth mentioning in the case of PCOS are the LH and the follicle-stimulating hormone (FSH). In the follicles, granulosa and theca cells are sites of action for FSH and LH gonadotropins, and sites for steroid hormone production. Interactions between theca and granulosa cells provide the base of the ovarian function. LH acts on the theca cells to promote the production of androgen from cholesterol that is then utilized by the granulosa cells to produce estrogen under the control of FSH [18]. FSH, on the other hand, stimulates the development and maturation of ovarian follicles.

In women with PCOS, it has been found that serum LH concentration is significantly increased, while the FSH value is decreased compared to healthy women, and this change eventually results in an elevated LH/FSH ratio [19]. Increased LH secretion has several consequences. The LH stimulation of ovarian theca cells drives the synthesis of testosterone; thus, elevated LH levels can lead to hyperandrogenism. Furthermore, elevated serum LH concentration is closely associated with a reduced chance of conception and an increased risk of miscarriage [20]. Through the HPG axis, LH release is a result of pulsatile GnRH release from the hypothalamus to the pituitary. A high pulse frequency of GnRH secretion favors the release of LH while a low frequency of GnRH secretion contributes to a greater FSH release [21]. Based on these hormonal changes, determination of the LH/FSH ratio serves as a gold standard in the assessment of PCOS and is used as a marker for ovarian reserve capacity [22].

### 2.2. Action of Physical Exercise on the HPG Axis Hormones in PCOS

Recent studies have shown that lifestyle modifications, including physical exercise, have protective effects on hormonal disturbances manifested in PCOS. There is no doubt about the role of hypothalamic pathologies in relation to PCOS. In this scenario, hypothalamic inflammation has been characterized as a potential pathophysiologic basis for the heterogeneity of clinical and hormonal presentation in PCOS [23]. Studies show that an imbalanced diet and obesity can target GnRH neurons in the hypothalamus, which integrates all signals in the brain to regulate reproduction. Due to their location in the brain, GnRH neurons are vulnerable to proinflammatory cytokines, immune cell infiltration, and microglia expansion; thus, hypothalamic pathologies result in neuroendocrine and consequent metabolic disturbances. However, physical exercise can induce defense mechanisms by decreasing microglia activation and by improving insulin sensitivity [24]. Consequently, reducing hyperinsulinemia results in a normo-androgenic pattern with decreased LH concentration.

Additionally, compelling new evidence indicates that the mechanism by which physical exercise improves neuroendocrine imbalance can be associated with an increased activity of the hypothalamic–pituitary–adrenal (HPA) axis. Consequently, the HPA axis orchestrates the release of glucocorticoids, which exert negative feedback control at the level of the hypothalamus/GnRH and the anterior pituitary gland/gonadotropins [25]. Similar to other non-communicable diseases, it is important to note that the duration and intensity of exercise as well as individual mental health (stress-related conditions) are major regulator factors in the regulatory loop of the HPA axis, cortisol, and reproduction [26]. Supporting this hypothesis, Bonab and Parvaneh provided findings on the effects of aerobic exercise in adolescent girls with PCOS. They found that 12-week aerobic exercise improved estrogen and testosterone levels as well as had a positive impact on lipid profile [27]. Jedel et al. proved that 16 weeks of aerobic exercise is an effective therapy for hyperandrogenism and oligo/amenorrhea [28].

### 2.3. Anti-Müllerian Hormone and PCOS

Another hormone used closely together with the LH/FSH ratio for identification and diagnostic purposes is the anti-Müllerian hormone (AMH). AMH is a product of granulosa cells of the preantral and small antral follicles in women. AMH regulates folliculogenesis by inhibiting the recruitment of follicles from the resting pool in order to select for the dominant follicle, after which the production of AMH diminishes [29]. Histomorphological features of PCOS can be characterized by more pre-antral and small follicles in the ovaries, partially due to hyperandrogenism, and more AMH is generally produced than in normal ovaries [30]. AMH has been shown to inhibit FSH-induced aromatase activity and abolishes the FSH growth-promoting effects on granulosa cells, which leads to a decreased estradiol production [29]. This imbalance suggests that increased AMH levels likely play a role in the causation of anovulation and PCOS [30].

### 2.4. Effects of Physical Exercise on Anti-Müllerian Hormone in PCOS

The correlation between AMH level and ovarian reserve function is well described. Recent studies also highlight that AMH and antral follicle count are the most sensitive parameters in the assessment of ovarian reserve capacity [31], which can be influenced by exercise [32]. Additionally, Al-Eisa et al. reported that 12 weeks of moderate aerobic exercise significantly reduced AMH levels, which was verified by transvaginal ultrasonographic examination with a result of improved follicle count [33]. Previous findings were verified by Wu et al., who concluded that 12-week aerobic exercise was able to improve ovarian reserves by diminishing AMH levels [34].

### 2.5. Insulin, Adipokines, and PCOS

Besides gonadotropins and AMH, there is a strong relationship between PCOS and pancreatic β-cell-secreted insulin release. During physiological processes, insulin activity plays a role in the function of the HPG axis and ovulation. Consequently, the development and manifestation of PCOS can be associated with insulin-related metabolic abnormalities, such as insulin resistance and compensatory hyperinsulinemia and vice versa. Although insulin resistance is a key feature of both obese and lean individuals, obesity can predispose to long-term endocrine–metabolic comorbidities. In a previous study, Cadagan et al. investigated the effects of insulin on theca cells and found that PCOS can disrupt steroid biosynthesis in ovarian theca cells, which is further augmented under hyperinsulinemia and increased LH secretion [35]. Insulin also stimulates ovarian androgen production in theca cells, which is different from the effects on glucose metabolism [36]. In turn, insulin resistance is affected and maintained by hyperandrogenism. The increased secretion of androgen is associated with the dysfunction of islets of Langerhans, thereby compromising the pancreatic metabolic functions and causing hyperinsulinemia. In preclinical studies, a direct relationship is revealed among the overexposure of androgens, hyperinsulinemia, insulin resistance, and type 2 diabetes mellitus in women with PCOS [37]. The altered endocrine–metabolic environment can be associated with adipose tissue accumulation, which can predispose PCOS-affected individuals to obesity. Although obesity in itself is a risk factor for numerous pathologies, morphological and functional alterations of adipose tissue result in a dysregulated adipokine secretion. A large number of adipokines are secreted from the adipose tissues, which have various impacts on insulin homeostasis. Among them, some adipokines mimic or induce insulin-like activity and stimulate insulin receptors, whereas others possess insulin-sensitizing effects. When the well-regulated adipokine secretion pattern is disrupted, the insulin metabolism is also disturbed, which can eventually aggravate the life expectancy of women with PCOS. Based on all these, adipokines are often used as biomarkers for PCOS-related insulin resistance [38,39].

### 2.6. Effects of Physical Exercise on Insulin Sensitivity and Adipokines in PCOS

The mechanism by which exercise results in a normo-androgenic environment is attributable to the involvement of the HPG axis. In this scenario, reduced hyperinsulinemia restores the sensitivity of the GnRH pulse activator role in diminishing LH release and androgen overproduction. These changes can eventually lead to dominant follicle maturation and regular ovulation [40]. Moreover, exercise improves glucose and insulin metabolisms by restoring glucose homeostasis through increased skeletal muscle glucose disposal [41]. The molecular background of the exercise-induced protective mechanism is that exercise activates the protein kinase C (PKC)/Akt/glucose transporter-4 (GLUT-4) signaling circuit that is damaged in PCOS [42,43]. Subsequently, the improvement in insulin sensitivity may therefore reduce inflammation and the release of cytokines that promote insulin resistance.

The exercise-induced reduction in abdominal obesity resulting from the metabolically active visceral adipocytes may also lead to reduced secretions of tumor necrosis factor-alpha (TNF-α) and IL-6 [44]. In addition to pro-inflammatory cytokines, adipokine release is also altered as a consequence of physical exercise. Among adipokines, the roles of leptin, resistin, vaspin, apelin, and adiponectin are highlighted in reproductive function and insulin sensitivity [38,45]. In a previous study, Al-Eisa et al. found that the change in AMH and adiponectin levels correlated significantly with physical activity level [33]. In agreement with these results, further studies have verified that the exercise-induced improvement in body fat percentage positively affects adipokine release [46,47].

### 2.7. IGF-1 and PCOS

In addition to the classic regulators such as FSH and LH, insulin-like growth factor (IGF) is involved in the regulation of insulin and androgens. IGF-1 plays a major role in ovarian tissue. IGF-1, once bound in ovarian granulosa cells, activates primordial follicles, enhances follicle development, and stimulates the proliferation and steroidogenesis of theca interstitial cells. Women with PCOS show an increase in the bioactivity of IGF-1, resulting from an increase in IGF-1 levels in non-obese patients or from a reduction in insulin-like growth factor-binding protein-1 (IGFBP-1) concentrations in obese patients. It was demonstrated by several research groups that the combined actions of IGF-1 along with growth hormone (GH) are responsible for the elevation in LH and the consequent hyperandrogenism in women with PCOS [48,49]. Increased IGF-1 levels can also lead to increased insulin sensitivity, which is strongly associated with obesity, as IGF-1 increases peripheral glucose uptake [50].

### 2.8. Effects of Physical Exercise on IGF-1 in PCOS

There have been a few studies on the link between physical exercise and IGF-1, and the effect of exercise on IGF-1, however, shows discordant results [51,52]. Besides these findings, evidence on the relationship between exercise and IGF-1 in PCOS is more limited. Stener-Victorin et al. did not detect any changes in IGF-1 level between control and trained individuals [53]. Szczuko et al. reported that dietary intervention, another lifestyle factor in PCOS therapy, increased IGF-1, and its concentration was correlated with the level of SHBG and HDL [54].

## 3. Role of Oxidative Stress and Inflammation in PCOS

### 3.1. Oxidative Stress and Lipid Peroxidation

With the keen awareness that PCOS is a major public health problem in women of reproductive age, understanding the underlying mechanisms driving PCOS is vital for devising targeted interventions and improving clinical outcomes. Oxidative stress as a general term is usually used to describe an imbalance between the production of free radicals and antioxidant defense mechanisms [55]. A large number of studies verify a causal relationship between oxidative stress and PCOS. A wide range of oxidative and antioxidant biomarkers serve as useful targets to estimate and evaluate the risk and role of oxidative damage in fertility-related conditions. Among oxidative biomarkers, malondialdehyde (MDA), nitric oxide (NO), advanced glycosylated end products (AGEs), and xanthine oxidase (XO) can be associated with increased oxidative stress in women with PCOS.

#### 3.1.1. MDA and PCOS

Murri et al. showed a meta-analysis which proved a ~47% increase in MDA concentration in women with PCOS compared to the healthy individuals [56]. MDA is a result of lipid peroxidation of polyunsaturated fatty acids and is among the most researched biomarkers, which lead to tissue damage through the disruption of protein structure and functions. Kuscu and Var compared blood MDA levels in PCOS patients with healthy controls and found that the MDA level was significantly higher in the PCOS group and was separate from obesity [57]. In accordance with these findings, Zhang et al. demonstrated that PCOS patients exhibited significantly increased serum MDA levels compared to the control group [58]. These results shed light on the fact that oxidative stress can affect the development and progression of PCOS, which can be independent from adipose tissue accumulation. NO is a free radical, and it is an important cellular signaling molecule involved in many pathological processes compromised in PCOS [55,59].

#### 3.1.2. Nitric Oxide and PCOS

In the last twenty years, the importance of NO in both the oxidative and inflammatory environment has been recognized. In the ovary, NO can be generated not only by ovarian cells but also by the ovarian vasculature and by the resident or infiltrating macrophages [60]. NO is generated by one of three nitric oxide synthase enzymes: endothelial NOS (eNOS), inducible NOS (iNOS), and neuronal NOS (nNOS). Nitric oxide is a freely diffusible molecule that possesses a key role in various reproductive and endocrine conditions, such as oocyte maturation, follicular development, ovulation, and PCOS-related cardiovascular complications. PCOS-related changes in NO levels are widely investigated; however, the role of NO in this condition is still a matter of debate. In a meta-analysis by Murri et al. [56], there was no statistically significant difference in plasma NO levels in women with PCOS compared with controls. Karabulut et al. researched the relationship between PCOS and oxidative stress levels by measuring NO. Their results showed statistically higher levels of NO in blood samples of PCOS patients compared to the control groups [61]. Nevertheless, recent findings suggest that the deficiency in NO in PCOS can be associated with the arrest of follicular development [59].

#### 3.1.3. AGEs, AOPPs, and PCOS

AGEs or ‘glycotoxins’ can be associated with the severity of oxidative damage and inflammation. They are also called the end products of a chemical process in which the carbonyl group of carbohydrates reacts non-enzymatically and interacts with lipids or with amino groups of proteins [62]. AGE levels as oxidative stress markers have been reported in several studies and evidence suggests that AGEs result in a hormonal imbalance in PCOS by altering enzyme functions and leading to inflammatory changes and insulin resistance [62]. Lin et al. evaluated the effect of AGEs on the function of granulosa cells and found that the proliferation of granulosa cells and the production of progesterone were inhibited by treatment with AGE products. These changes occurred through downregulation of the LH receptor/cAMP regulatory activity [63]. Advanced oxidation protein products (AOPPs) are novel markers of oxidant-mediated protein damage and they act as a unique class of proinflammatory mediators [55]. Hyderali et al. summarizes that plasma levels of AOPPs were significantly higher in women with PCOS compared with the healthy controls [64].

#### 3.1.4. Xantin Oxidase in PCOS

Another oxidative biomarker associated with PCOS is xanthine oxidase. XO is an enzyme that participates in the generation of superoxide anion radicals. Serum XO, which plays an important role in the catabolism of purines in humans and generates ROS, was increased in PCOS in studies [55]. In a cross-sectional study, Isik et al. examined the relationship among XO, oxidative stress, inflammation, and blood parameters in women with PCOS. They concluded a positive correlation between XO activity and LH/FSH ratio as well as inflammatory status [65].

#### 3.1.5. Mitochondrial DNA and PCOS

Increased ROS production can induce damage in mitochondrial components such as mtDNA, proteins, and lipids and finally prompts cell apoptosis mediated by mitochondrial alterations [66]. In a previous study, Zeber-Lubecka et al. summarized that any defects at the level of mtDNA replication affect the formation of numerous mutations; thus, mtDNA damage can be a contributor in the pathogenesis of PCOS. They demonstrated that mitochondrial mutations can lead to impaired oxidative phosphorylation, disruption to insulin signaling pathways, and result in an increased ROS production and a consequent oocyte impairment [67]. Sang-Hea Lee et al. have demonstrated that the number of mtDNA copies were found to be lower in women with PCOS than in the control groups [68], with a negative correlation with the severity of the syndrome. These results are verified by the findings of Zhang et al., who also demonstrated that decreased mtDNA content in peripheral leukocytes is associated with the development of T2DM, which is the late-stage complication of PCOS [69].

### 3.2. Effects of Physical Exercise on Oxidative Stress Parameters in PCOS

It is well documented that in many non-communicable diseases such as PCOS, there is a strong relationship between ROS generation and glucose/insulin metabolism, which can be characterized by a dysregulated mitochondrial function and insulin resistance [70]. The main causes for these pathological changes are an incomplete oxidation of fatty acids, resulting in lipid accumulation, which may inhibit insulin signaling, and an increased ROS content and oxidative stress, potentially resulting in mitophagy and apoptosis. Consequently, physical exercise has a beneficial effect on these pathways. The above-mentioned physiological changes were also verified by Malamouli et al., who concluded that exercise intervention has a positive impact on mitochondrial health in the skeletal muscle of women with PCOS. These changes were mediated by decreasing ROS production along with improving insulin sensitivity [71].

### 3.3. Antioxidant Defense Mechanisms

A large number of studies have underpinned that shifts in the oxidant/antioxidant homeostasis can maintain or aggravate the PCOS-related redox imbalance. In the complex biochemical background of PCOS pathologies, there are key antioxidants that have an influence on the endocrine disturbance, including SOD, GPx, GSH, as well as nuclear factor erythroid 2-related factor 2 (Nrf2).

#### 3.3.1. Superoxide Dismutase and PCOS

SOD is an enzyme and an integral part of the antioxidant defense system that eliminates superoxide anions (O^2−^), a major oxygen radical, by catalyzing them into H_2_O_2_, which is eventually turned into water by GPx. Depending on the metal cofactor, SOD can have several variants and protein folds, such as the Cu/Zn type, Fe and Mn type, and Ni type [72]. SOD activity in PCOS is reported in several studies; however, the results are controversial. In a previous study, Seleem et al. investigated the role of oxidative stress and inflammation in PCOS and found that both follicular fluid and serum SOD levels were significantly lower in women with PCOS compared to the control group. The mean relative levels of Cu and Zn SOD mRNAs were also significantly lower in cells isolated from the follicular fluid in PCOS than the control group [73]. Sabuncu et al. showed that women with PCOS had higher SOD levels than healthy individuals [74], while in another study, Zhang et al. established a contrary change [58]. In accordance with Zhang et al., the study by Bizoń et al. further supported that SOD activity significantly decreased in both serum and follicular fluid and suggested that changes in SOD activity could be a clinical parameter for determining systemic oxidative stress in PCOS [75].

#### 3.3.2. Glutathione Peroxidase, Glutathione, and PCOS

Continuing the list of the antioxidants, GPx is an enzyme family that protects the organism from oxidative damage by reducing lipid hydroperoxides to their corresponding alcohols and reducing H_2_O_2_ to water. The GPx activity evaluation for antioxidant defense assessment in PCOS was reported in a large number of studies [56]. Results regarding GPx activity are still conflicting; thus, some studies show elevated GPx values in women with PCOS or demonstrate a similar GPx range between PCOS patients and healthy individuals. Sulaiman et al. concluded that the increase in GPx activity can be associated with the fact that GPx has a higher affinity toward peroxides and thus scavenge free radicals more efficiently [8]. Contrary to these findings, there are previous findings which demonstrated a GPx reduction in PCOS. Uckan et al. concluded that oxidative stress and decreased antioxidant parameters (e.g., SOD, catalase, and GPx) in PCOS patients were correlated with hyperinsulinemia, hypertension, and dyslipidemia [76]. Similar to GPx, GSH is another major antioxidant that can prevent damage to cellular components against reactive oxygen species, free radicals, peroxides, and heavy metals. Sulaiman et al. demonstrated a lower GSH level in women with PCOS compared to healthy women [8]. These results support the conclusions reached by other researchers, such as Chelchowska et al., whose findings showed a reduction of up to 50% in glutathione levels in women with PCOS compared to healthy individuals [77].

#### 3.3.3. Nrf2 and PCOS

Nrf2 is a master transcriptional factor that regulates the expression of antioxidant proteins to protect against oxidative damage and inflammation. After activation, Nrf2 detaches from Keap1 and translocates to the nucleus to activate several antioxidant genes, such SOD, catalase, heme oxygenase-1, and GPx. In patients with PCOS, an association has been reported between reduced Nrf2 cell content and hyperandrogenism, insulin resistance, and obesity [78]. Wang et al. examined the role of the Keap1/Nrf2 pathway on oxidative stress in the ovaries of women with PCOS as well as an in vitro human ovarian granulosa cell line. As a result of oxidative stress, they detected an increase in Nrf2 expression and concluded that in the early stage of oxidative stress, the body adaptively increases Nrf2 expression to maintain the functionality of ROS removal. However, during sustained oxidative stress leading to cell damage, antioxidant function is further impaired, which results in a decrease in Nrf2 expression. In agreement with other studies, they verified that antioxidant and anti-inflammatory supplementation can up-regulate and thus ensure the defense mechanism, Nrf2, and increase the antioxidant enzyme concentrations [79,80].

### 3.4. Effects of Physical Exercise on Antioxidant Parameters in PCOS

Although there are few studies which examine the effects of physical exercise on the antioxidant parameters, specifically with the mechanism of action in women with PCOS, the beneficial role of exercise in reproductive health is undeniable and unquestionable. As seen in many types of pathological conditions, there is no standard template for the relationship between training and ROS production, which determine the consequent antioxidant defense mechanism. The extent to which ROS is helpful or harmful depends on the duration and intensity of exercise as well as the fitness condition and nutritional status of the individual [41,81]. With a previous study, Wu et al. support the protective effect of exercise in PCOS. They reported that a 12-week aerobic training period in women with PCOS increased SOD levels in line with total antioxidant capacity, while MDA levels were reduced and AMH was improved [34].

### 3.5. Inflammation

Compelling evidence suggests that hyperandrogenism-associated immune-stress and an inflammatory environment can lead to reproductive dysfunction via the disturbance of the HPG axis [82,83,84]. A large number of studies underpin that certain types of immune cells are involved in PCOS pathogenesis. The most abundant immune cells in PCOS are macrophages and neutrophils. These cells produce inflammatory cytokines, such as IL-1, IL-6, IL-18, and TNF-ɑ, which are present in high levels in both the serum and follicular fluid of women with PCOS. When discussing the effect of the immune system on PCOS, it is important to note that natural killer cells, dendritic cells, lymphoid T and B cells, and their role in cytokine dysregulation are also key features in PCOS [85]. T cells, which are classified into helper T cells, cytotoxic T cells, and regulatory T cells, participate in the regulation of ovarian homeostasis. Therefore, even a slight disturbance in the ratio of T cells can result in a proinflammatory phenotype with high levels of cytokines, including TNF-α, IL-6, interferon-γ (IFNγ), and IL-17, which can be associated with PCOS, preeclampsia, or infertility [86]. Although the link between T cells and PCOS is well documented, the role of B cells is not fully elucidated. In a very recent study, Ascani et al. showed that B cells are not central mediators of PCOS pathology and that their frequencies are altered as a direct effect of androgen receptor activation. Consequently, hyperandrogenic women with PCOS have increased frequencies of double-negative B memory cells and increased levels of circulating immunoglobulin M [87].

Based on all these, hyperandrogenism-induced immune system stimulation in PCOS can change the proinflammatory cell profile, leading to a disturbed cytokine release.

#### 3.5.1. Interleukins and PCOS

Among interleukins, IL-6 acts as a pro-inflammatory cytokine, and the production of the IL-6-producing T cells, neutrophils, and M1 macrophages is strongly correlated with hyperandrogenism. Additionally, IL-6 is produced by adipocytes and is thought to be a reason why obese individuals have higher levels of inflammatory parameters, such as C-reactive protein (CRP) [88,89,90]. Previously, Fulghesu et al. underpinned that women with PCOS exhibited higher IL-6 values compared to healthy individuals, and this pathological elevation was further enhanced as a consequence of insulin resistance [91].

IL-1 and IL-18 are members of the IL-1 cytokine superfamily and are produced primarily from monocytes and macrophages. IL-1α and IL-1β directly affect progesterone and estradiol production in cultures of purified human granulosa cells [92]. IL-1α inhibits estradiol production of the granulosa cells, while IL-1β enhances basal progesterone secretion of human granulosa and theca cells and in small and large follicles [93]. Zangeneh et al. concluded that increased IL-1α can impair the feedback system of the neuro-inflammation process and that increased IL-1β can be associated with anovulation in women with PCOS [94,95]. Interleukin 18 (IL-18) is a cytokine with a relatively late discovery that was initially described as an IFNγ-inducing factor. Elevated IL-18 levels were already observed in several low-grade inflammatory conditions, such as obesity or prediabetes, but their importance within PCOS is controversial. Yang et al. examined changes in IL-18 levels in women with PCOS and they detected increased IL-18 levels in women with PCOS. Furthermore, IL-18 values showed a positive correlation with obesity, IR, and hyperandrogenism [96]. By contrast, Kabakchieva et al. demonstrated no significant difference in IL-18 levels between healthy women and those with PCOS [97]. Numerous studies found a relationship between IL-18 levels and obesity, but not between IL-18 and PCOS specifically [98,99].

#### 3.5.2. TNF-α and PCOS

Continuing the list of inflammatory parameters, TNF-α is a cytokine that has pleiotropic effects on various cells, including muscles, adipose tissues, macrophages, and ovaries. TNF-α is mainly generated by activated macrophages, T-lymphocytes, and natural killer cells [100]. An inappropriate or excess activation of TNF-α signaling is associated with chronic inflammation and can eventually lead to the development of pathological complications as it is functionally known to trigger a series of various inflammatory molecules, including other cytokines and chemokines [100]. TNF-α promotes insulin resistance, causes hyperandrogenism, and is involved in follicular development; hence, it has been implicated in the pathophysiology of PCOS. Thathapudi et al. investigated TNF-α levels and found that its concentration was significantly increased in women with PCOS [101]. In a comprehensive meta-analysis by Gao et al., the results clearly showed that TNF-α levels were significantly increased in PCOS, which was directly related to the insulin resistance and androgen excess [102]. Oróstica et al. also demonstrated that increased TNF-α signaling negatively affects the glucose uptake of the endometrial stromal cells [103].

#### 3.5.3. NF-κB and PCOS

NF-κB is a protein complex which controls cytokine production and cell survival and is involved in cellular responses to stimuli such as stress, cytokines, and free radicals. In PCOS, hyperglycemia-induced inflammation can directly stimulate hyperandrogenism. This is suggested by the direct correlation of the plasma levels of testosterone or androstenedione with p65 expression and intranuclear NF-κB. Physiological hyperglycemia induced by insulin resistance results in an increased NF-κB level in women with PCOS, and vice versa [104]. In another study, González et al. demonstrated that plasma levels of testosterone and androstenedione positively correlated with the percent change of NF-κB, leading to elevated levels of relevant inflammatory indicators, which may serve as major contributors in the pathogenesis of PCOS [105].

### 3.6. Effects of Physical Exercise on Inflammatory Parameters in PCOS

Since chronic low-grade inflammation is a known key contributor to PCOS, the effects of exercise, specifically aerobic exercise, were analyzed on the most common inflammatory markers, such as IL-6, TNF, and CRP. One such study was conducted by Elbandrawy et al., who concluded that aerobic exercise is effective in lowering IL-6, TNF-α, and CRP in women with PCOS [106]. Some of the possible mechanisms that explain the effect of exercise on inflammation include the exercise-stimulated accumulation of anti-inflammatory cytokines (such as IL-10 and IL-1 receptor antagonist), alterations in psychosocial factors (depression, stress, and anxiety), and weight loss via reducing the visceral fat amount [107]. Based on all this information, aerobic exercise is beneficial in the management of PCOS and is indicated as an effective modality in the prevention and therapy of PCOS.

## 4. Conclusions

**Figure 2:**
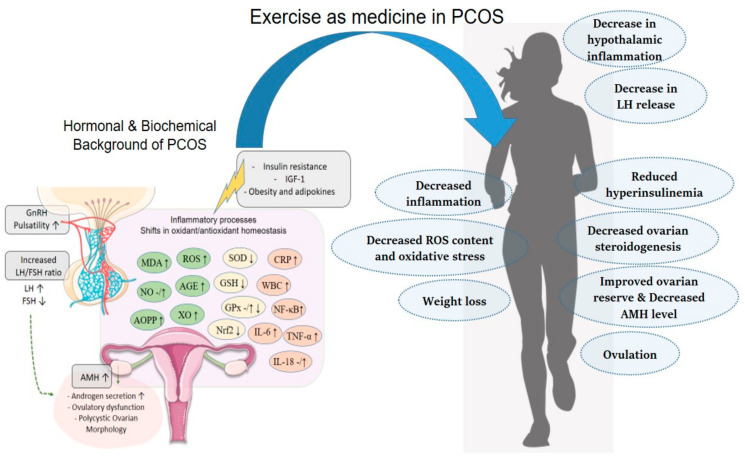
Exercise-induced normo-androgenic environment via decrease in inflammatory and oxidative stress parameters in women with PCOS. GnRH: gonadotropin-releasing hormone; LH: luteinizing hormone; FSH: follicle-stimulating hormone; AMH: anti-Müllerian hormone; MDA: malondialdehyde; NO: nitric oxide; ROS: reactive oxygen species; AGE: advanced glycosylated end product; AOPP: advanced oxidation protein product; XO: xanthine oxidase; SOD: superoxide dismutase; GSH: glutathione; GPx: glutathione peroxidase; Nrf2: nuclear factor erythroid 2-related factor 2; CRP: C-reactive protein; WBC: white blood cell; NF-κB: nuclear factor-kappa B; IL-6: interleukin-6, IL-18: interleukin-18; TNF-α: tumor necrosis factor-alpha, IGF-1: insulin-like growth factor-1.

Despite a long history of studies on PCOS, there are still many unanswered and controversial questions for researchers and clinicians. Lifestyle changes (physical exercise, diet, and behavioral changes) serve as first-line management in international evidence-based guidelines for PCOS. Our review article provides a detailed insight into the protective effects of physical exercise on hormonal, inflammatory, and oxidative/antioxidant processes in PCOS. The mechanisms by which exercise improves PCOS are related to decreased hyperinsulinemia and a balanced androgenic environment, with an underlying reduction in inflammatory processes and improvement in oxidant/antioxidant homeostasis. The take-home message seems feasible: exercise to protect your reproductive health, which is a cornerstone of life quality and well-being (Figure 2).
